# Flexible, Porous, and Metal–Heteroatom-Doped Carbon Nanofibers as Efficient ORR Electrocatalysts for Zn–Air Battery

**DOI:** 10.1007/s40820-019-0238-4

**Published:** 2019-01-19

**Authors:** Qijian Niu, Binling Chen, Junxia Guo, Jun Nie, Xindong Guo, Guiping Ma

**Affiliations:** 1 Key Laboratory of carbon Fiber and Functional Polymers, Ministry of Education, Beijing University of Chemical Technology, Beijing, 100029 People’s Republic of China; 2 State Key Laboratory of Chemical Resource Engineering, Beijing University of Chemical Technology, Beijing, 100029 People’s Republic of China; 3 College of Engineering, Mathematics and Physical Sciences, University of Exeter, Exeter, EX4 4QF UK

**Keywords:** Electrospinning, Zn/Co-ZIFs, Carbon nanofibers, Flexible porous structure, ORR, Zn–air battery

## Abstract

Developing an efficient and durable oxygen reduction electrocatalyst is critical for clean-energy technology, such as fuel cells and metal–air batteries. In this study, we developed a facile strategy for the preparation of flexible, porous, and well-dispersed metal–heteroatom-doped carbon nanofibers by direct carbonization of electrospun Zn/Co-ZIFs/PAN nanofibers (Zn/Co-ZIFs/PAN). The obtained Zn/Co and N co-doped porous carbon nanofibers carbonized at 800 °C (Zn/Co–N@PCNFs-800) presented a good flexibility, a continuous porous structure, and a superior oxygen reduction reaction (ORR) catalytic activity to that of commercial 20 wt% Pt/C, in terms of its onset potential (0.98 V vs. RHE), half-wave potential (0.89 V vs. RHE), and limiting current density (− 5.26 mA cm^−2^). In addition, we tested the suitability and durability of Zn/Co–N@PCNFs-800 as the oxygen cathode for a rechargeable Zn–air battery. The prepared Zn–air batteries exhibited a higher power density (83.5 mW cm^−2^), a higher specific capacity (640.3 mAh g^−1^), an excellent reversibility, and a better cycling life than the commercial 20 wt% Pt/C + RuO_2_ catalysts. This design strategy of flexible porous non-precious metal-doped ORR electrocatalysts obtained from electrospun ZIFs/polymer nanofibers could be extended to fabricate other novel, stable, and easy-to-use multi-functional electrocatalysts for clean-energy technology.

**Electronic supplementary material:**

The online version of this article (doi:10.1007/s40820-019-0238-4) contains supplementary material, which is available to authorized users.

## Highlights

- Doping and porosity generation were completed simultaneously.
- Metal–heteroatom-doped carbon nanofibers are flexible, porous, and well dispersed.
- Results include excellent oxygen reduction reaction and enhanced Zn–air battery performance.

**Electronic supplementary material:**

The online version of this article (doi:10.1007/s40820-019-0238-4) contains supplementary material, which is available to authorized users.

## Graphical abstract

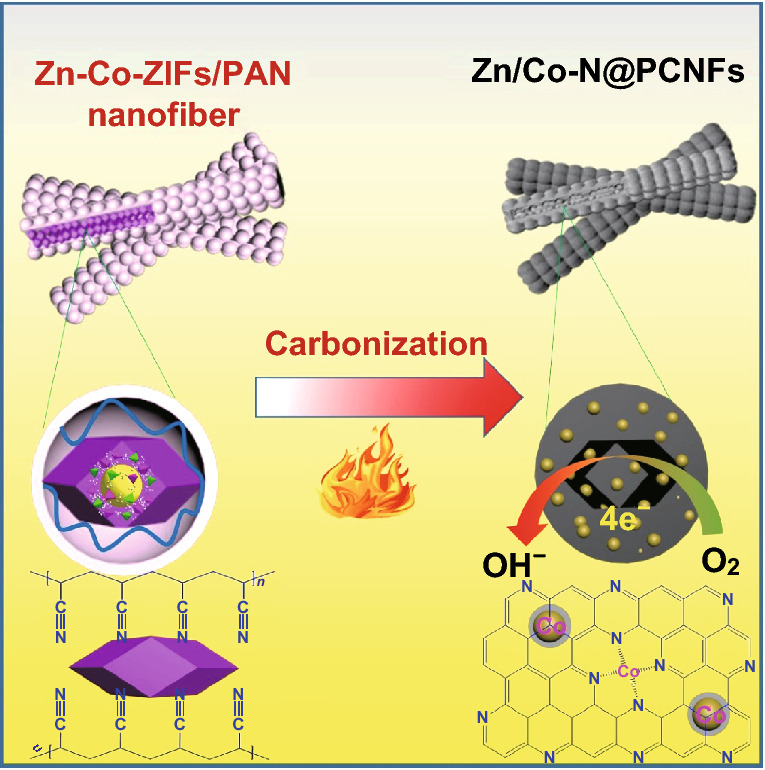

## Introduction

New energy technology has become an optimal solution for the energy crisis and environmental pollution caused by the rapid depletion of fossil resources [1, 2]. Recently, sustainable energy conversion and storage systems, such as supercapacitors, fuel cells, and batteries, have been developed rapidly. Among these various new energy devices, fuel cells and metal–air batteries have received increasing attention because of their low contribution to pollution. However, the oxygen reduction reaction (ORR), one of the key reactions of fuel cells and metal–air batteries, has sluggish intrinsic electrode kinetics, hampering the practical application of fuel cells and metal–air batteries [3]. Up to now, Pt-based materials are known as the best catalytic materials for ORR. However, these materials suffer from the prohibitive cost, severe scarcity, serious intermediate tolerance, and poor stability [4]. As an alternative, non-precious metal-doped carbon-based materials with various nanostructures, such as porous/hollow carbon nanoparticles [5–7], porous/core–shell carbon nanofibers [8–10], porous/sandwich-type graphene nanosheets [11–13], and porous graphene aerogels [14, 15], have emerged and attracted great attention. Porous carbon materials, containing catalytic active metal nanoparticles for effective catalysis, have been regarded as crucial supporting materials, owing to their high specific surface area, highly porous structure, and excellent electrical conductivity.

Metal–organic frameworks (MOFs), constructed by bridging metal ions and organic functional ligands into three-dimensional (3D) ordered crystal frameworks with rich micropores and high surface areas, provide a good platform for designing metal–heteroatom-doped carbon catalysts [16, 17]. The first MOF used as a template for porous carbon synthesis was reported by Xu et al. [18]. Among the different types of MOFs, zeolitic imidazole frameworks (ZIFs), a subclass of MOFs, are the most-studied candidates because of their high content of nitrogen and metal ions [19]. Moreover, such complex units consisting of nitrogen and metal ions (MN_4_) are easy to form active sites for ORR. Thus, N-rich ZIFs (e.g., ZIF-8, ZIF-67, bimetallic Zn/Co-ZIFs) were used as self-sacrificing templates and precursors to construct electrocatalysts with high surface areas, uniform N doping, and Co–N_*x*_ active sites by the high-temperature carbonization [20]. However, there remain some problems associated with the obtained ZIF-derived electrocatalysts, such as poor electrical conductivity, aggregation of loaded metal nanoparticles, and poor mechanical stability, which may affect their practical applications.

Recently, combining ZIFs with low-dimensional materials has gotten an increasing amount of attention. Tellurium nanowire-directed templating synthesis of ZIF-8 nanofibers has been demonstrated by Wang Zhang et al. [21]. After carbonization, the as-obtained ZIF-8 nanofibers can be easily converted into highly porous carbon nanofibers with complex network structures, hierarchical pores, and high surface areas, which are beneficial to the improvement of electrochemical properties. Ahn et al. [22, 23] reported a similar synthesis of one-dimensional (1D) hierarchically porous N- and Co-doped carbon nanotubes for efficient ORR by combining a 1D tellurium nanotube as the main template for the carbon nanotube backbone, with an anchored Zn/Co-ZIF as a sub-template for the carbon framework. The porous carbon derived from the bimetallic composites of ZIF-8 and ZIF-67, with a proper ratio, generates synergistic effects, such as a high degree of graphitic carbon, a formation of Co*–*N_*x*_ active sites, and a high surface area. To improve the inter-particle conductivity of the electrocatalysts, multiwall carbon nanotubes (MWCTs) were used in ZIF synthesis, which interconnect the nanoparticles and provide electron conducting highways. Zhang et al. [24] introduced MWCNTs to increase the electronic conductivity and mass transport of ORR catalysts derived from bimetallic Zn/Fe-ZIFs. ZnO nanorods and nanowires were also used as facile self-sacrifice templates to fabricate hierarchically porous carbon nanotubes from core–shell ZnO@ZIF-8 nanorods and ZnO@Zn/Co-ZIFs nanowires [25, 26]. The in situ reduction and evaporation of ZnO effectively resolved the aggregation issue during carbonization and therefore formed hierarchical pores without using any extra template. 1D carbon nanofiber materials have been paid extensive attention due to their excellent conductivity and flexibility, which are beneficial to improving their catalytic performance and designing flexible electronic devices [27, 28]. Moreover, carbon nanofibers not only solve the above-mentioned waste of inorganic templates but also provide longer electron transport channels. Electrospinning is a simple and efficient method for the preparation of nanofibers [29, 30]. Direct carbonization of electrospun precursor nanofibers is a fast and efficient method for preparing carbon nanofibers [31, 32]. Carbon nanofibers obtained via electrospinning followed by a subsequent carbonization have several advantages: (1) high electrical conductivity through the connection between the nanofibers, (2) fast mass transmission from the network structure and high surface area, and (3) cost effectiveness from the simple preparation procedure [33]. In our previous work, Zn/Co-ZIFs/PAN core–shell nanofibers were well-designed and prepared through Zn/Co-ZIFs grown in situ on the surface of electrospun nanofibers [34]. The results showed that electrochemical performance was improved. However, the electrochemical performance was still worse than that of the commercial catalyst, which may be due to its small surface area. Recently, Liu et al. [35] developed a novel N, Co-contained MOF-based hierarchical carbon nanofiber as an ORR catalyst, which was synthesized by incorporating Zn/Co-ZIFs with electrospun Co^2+^/PAN nanofibers, followed by carbonization and acid-leaching treatment. However, the size of the prepared ZIF nanocrystals is ultra-small in their study, and additional metal ions (Co^2+^) were required for the electrospinning process. Based on these studies, we investigated combining electrospun PAN nanofibers with just as-prepared Zn/Co-ZIFs in different contents, as a precursor for flexible, porous, and well-dispersed metal–heteroatom-doped carbon nanofiber catalysts.

Herein, we report a facile approach to prepare well-dispersed metal (Zn/Co) and heteroatom (N) co-doped porous carbon nanofibers (Zn/Co–N@PCNFs) film based on electrospun Zn/Co-ZIFs/PAN nanofibers. During the process, Zn/Co-ZIF nanocrystals with a larger size (~ 900 nm) and different contents were loaded onto electrospun PAN nanofiber without any additional metal ions. Such a facile method not only can yield a hierarchical porous structure but can also achieve a good distribution of metal active sites in the porous carbon nanofibers, which is important for ORR. Zn/Co–N@PCNFs-800 (carbonization temperature is 800 °C) exhibited an excellent ORR performance. In addition, the suitability and durability of Zn/Co–N@PCNFs-800 were tested as the oxygen cathode for primary and rechargeable Zn–air batteries, showing relatively good electrochemical properties.

## Experimental Section

### Materials

Polyacrylonitrile (PAN, *M*_w_ = 150,000 g mol^−1^), zinc nitrate hexahydrate (Zn(NO_3_)_2_·6H_2_O), cobalt nitrate hexahydrate (Co(NO_3_)_2_·6H_2_O), 2-methylimidazole (C_4_H_6_N_2_, MIM), methanol (MeOH), ethanol (EtOH, ≥ 99.7%), potassium hydroxide (KOH, 98%), and *N*,*N*-dimethylformamide (DMF) were all purchased from Aladdin Chemical Reagent Co. Nafion solution (5 wt%) was purchased from DuPont Co. Common commercial 20 wt% Pt/C catalyst and RuO_2_ were bought from Johnson Matthey Co. All chemicals were of analytical grade and used without further purification.

### Preparation of the Samples

#### Preparation of Zn/Co-ZIF Nanocrystals

The preparation of Zn/Co-ZIF nanocrystals was based on a previous procedure with modifications [36]. Typically, 5.0 mmol Zn(NO_3_)_2_·6H_2_O and 10.0 mmol Co(NO_3_)_2_·6H_2_O were dissolved into 150 mL methanol to form a clear solution. The molar ratio of Zn^2+^/Co^2+^ was set to 1/2. A mixture of 60 mmol 2-methylimidazole with 50 mL methanol was added to the above solution with 12 h incubation at room temperature. The product was separated by centrifugation and then washed thoroughly with methanol three times, and finally dried overnight at 60 °C under a vacuum oven.

#### Preparation of the Zn/Co-ZIFs/PAN Precursor Nanofibers

The Zn/Co-ZIFs/PAN precursor nanofibers were prepared by electrospinning [37]. In a typical experiment, 0.5 g PAN powder was dissolved into 4.5 g DMF solvent [38]. The blended solution was continuously stirred for 6 h at 40 °C. Then, 1.0 g Zn/Co-ZIF nanoparticles were added into above solution and stirred for another 6 h at 40 °C. Afterward, the electrospinning process was carried out with a high voltage of 20 kV and an extrusion rate of 0.6 mL h^−1^. The obtained nanofibers were collected on aluminum foil (~ 15 × 15 cm^2^). The collect distance between the nozzle and the aluminum foil was 15 cm. The Zn/Co-ZIFs/PAN nanofiber film was easily peeled off from the collector and put into a vacuum oven overnight at a temperature of 80 °C to remove the residual solvents.

#### Preparation of the Zn/Co–N@PCNF Electrocatalysts from Zn/Co-ZIFs/PAN Nanofibers

The obtained Zn/Co-ZIFs/PAN nanofiber film was pre-oxidized at 280 °C for 2 h at a heating rate of 2 °C min^−1^ under air atmosphere. The obtained pre-oxidized nanofiber film was then directly carbonized at the target temperatures (500, 600, 700, 800, 900, and 1000 °C) for 2 h at a heating rate of 5 °C min^−1^ in N_2_ atmosphere and then naturally cooled to room temperature to obtain the flexible, porous, and well-dispersed metal–heteroatom-doped carbon nanofibers. (The samples were named as Zn/Co–N@PCNFs-T, where T is the target carbonization temperature.)

### Physical Characterizations

The microstructure and surface morphology of the obtained samples were observed by scanning electron microscopy (SEM, S-4700, Hitachi, Japan). The internal structure and graphitic structure were investigated by transmission electron microscopy (TEM, Tecnai G2 T20, FEI, USA) and high-resolution transmission electron microscopy (HR-TEM, JEM 3010, JEOL, Japan). Scanning transmission electron microscopy (STEM) and color mapping were employed to distinguish the elemental dispersion in these samples by HR-TEM. The thermal decomposition behavior of the precursor nanofibers was determined by thermal gravimetric analysis (TGA, Q500, TA Instruments, USA). Fourier transform infrared (FT-IR) spectra of the samples were measured by spectrometer (Nicolet-is5 IR, Thermo Fisher Scientific, USA). The crystal structure of the samples was evaluated on a powder X-ray diffraction (XRD, D8 Advance, Bruker, Germany) system with Cu-Kα radiation. Raman spectra analysis was conducted on a Raman spectrometer (Invia Reflex, Renishaw, British) at 514 nm. X-ray photoelectron spectroscopy (XPS, Thermal Scientific K-Alpha XPS spectrometer) was employed to analyze the chemical composition of these samples. Nitrogen absorption/desorption isotherms were obtained on a Quantachrome Autosorb-iQ gas sorptometer via the conventional volumetric technique, and the corresponding surface areas were determined by using the Brunauer–Emmett–Teller (BET) method.

### Electrochemical Measurements

All electrochemical measurements were performed in a three-electrode system on an electrochemical workstation (CHI 760E, Shanghai Chenhua, China) in 0.1 M KOH electrolyte. A glassy carbon (GC) rotating disk electrode (RDE, ALS, Japan) of 4.0 mm in diameter was used as a working electrode. Before use, the working electrode was polished carefully with 50 nm Al_2_O_3_ powders to obtain a mirror-like surface and then washed with deionized water and ethanol and allowed to dry. A platinum wire and Ag/AgCl (3.0 M KCl) electrode were used as the counter and reference electrodes, respectively. The electrochemical measurements were carried out in a 0.1 mol L^−1^ KOH aqueous electrolyte at the temperature of 298 K. To prepare the working electrode, 5.0 mg of the catalyst was dispersed in a solution consisting of 1.0 mL of absolute ethanol and 100 µL of 5 wt% Nafion, and then sonicated for 1 h to form a well-dispersed black catalyst ink. For the catalyst ink, 5.0 µL was drop-cast onto the glassy carbon surface (~ 0.18 mg cm^−2^ loading) and dried at room temperature for electrochemical testing. The working electrodes were scanned for about 50 cycles until the signals were stabilized, and then, the data were collected. Before testing, a continuous N_2_/O_2_ flow was bubbled into the electrolyte for 30 min. The cyclic voltammetry (CV) experiments were cycled in 0.1 M N_2_- and O_2_-saturated KOH electrolyte solutions with a sweep rate of 50 mV s^−1^. The RDE tests were measured in 0.1 M O_2_-saturated KOH electrolyte solutions with a sweep rate of 10 mV s^−1^ and different speed rates (400–2500 rpm). For comparison, 20 wt% Pt/C was used in the same electrochemical tests.

For the ORR on an RDE, the electron transfer numbers can be calculated with Koutecky–Levich equations (Eqs. 1–3):

1 $$\frac{1}{j} = \frac{1}{{j_{\text{L}} }} + \frac{1}{{j_{\text{K}} }} = \frac{1}{{B\omega^{1/2} }} + \frac{1}{{j_{\text{K}} }}$$

2 $$j_{k } = nFkC_{0}$$

3 $$B = 0.2nFC_{0} \left( {D_{0} } \right)^{2/3} V^{ - 1/6}$$

where *j* is the measured current density; *j*_K_ and *j*_L_ are the kinetic and diffusion-limiting current densities, respectively; *ω* is rotation speed (rpm); *n* represents the electron transfer number in the oxygen reduction reaction; *F* is the Faraday constant (*F* = 96,485 C mol^−1^); *C*_0_ is the bulk concentration of O_2_ (1.2 × 10^−6^ mol cm^−3^); *D*_0_ is the diffusion coefficient of O_2_ in 0.1-M KOH electrolyte (1.9 × 10^−5^ cm^2^ s^−1^); *V* is the kinematic viscosity for electrolyte (0.01 cm^−2^ s^−1^); and *k* is the electron transfer rate constant. All potentials in this study were converted to the RHE reference scale using *E* (vs. RHE) = *E* (vs. Ag/AgCl) + 0.21 V + 0.0591 × pH.

Zn–air battery assembly and test: the rechargeable Zn–air battery performance was tested using a homemade Zn–air battery. To assemble the Zn–air battery, a polished zinc plate (0.3 mm of thickness) was used as the anode; an air electrode coated by 100 μL catalyst ink of Zn/Co–N@PCNFs-800 or a mixture of 20 wt% Pt/C + RuO_2_ (1:1 in a mass ratio) onto carbon paper (electrode area: 0.8 cm in diameter; catalyst loading: 1.2 mg cm^−2^), dried naturally to form a uniform catalyst layer, was used as the cathode; and 6.0 M KOH solution served as the electrolyte. The potential–current polarization curves for the batteries were recorded on a CHI 760e workstation. The discharge/charge performance and stability for the batteries were analyzed by a Lanhe-CT2001A testing system at room temperature [39].

## Results and Discussion

**Fig. 1:**
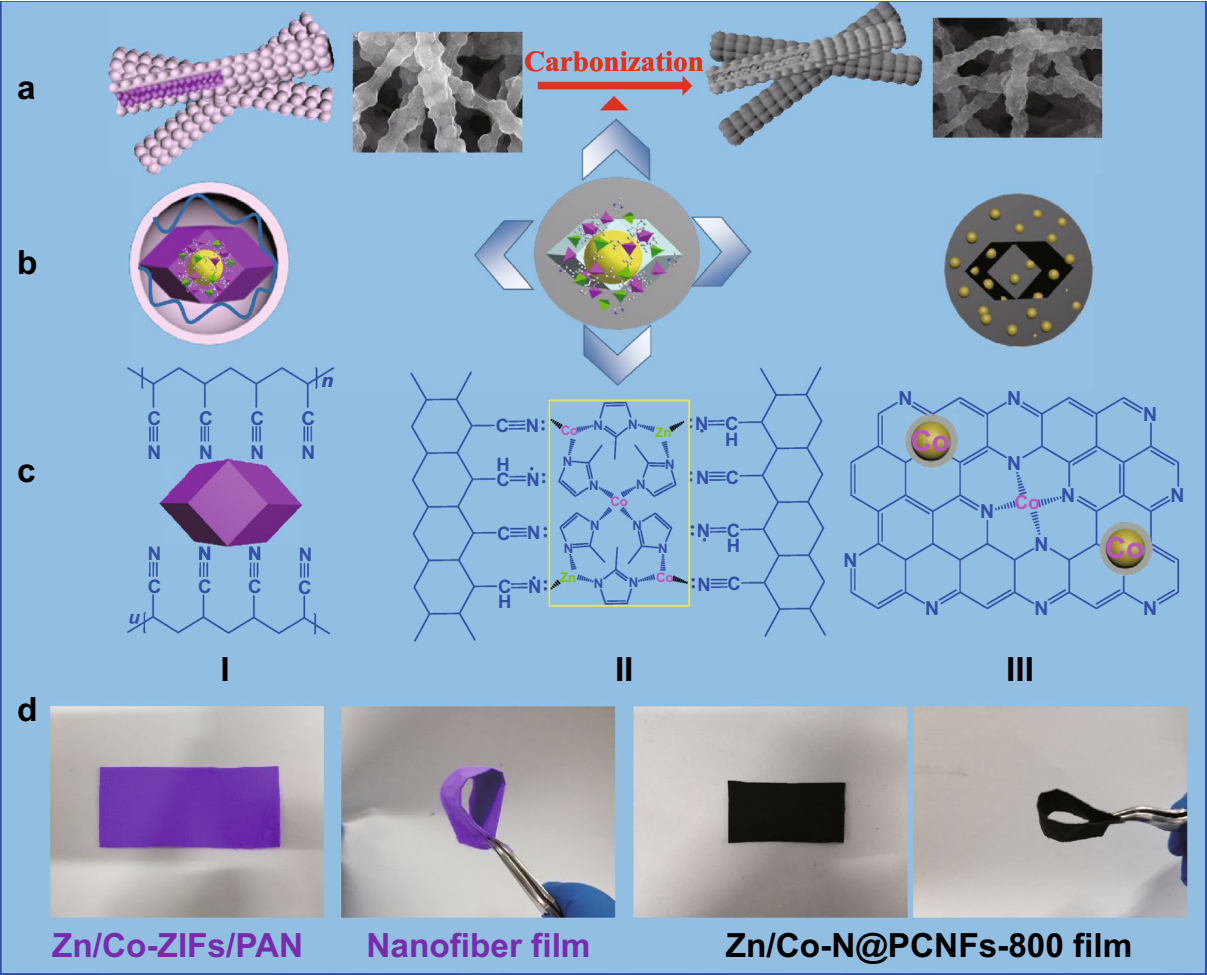
Schematic illustration for the preparation of Zn/Co–N@PCNFs from electrospun Zn/Co-ZIFs/PAN nanofibers. **a** The micro-morphologies of the nanofibers before and after carbonization. **b** The simulated cross section diagram. **c** The simulated molecular structure diagram. **d** The digital photographs of the nanofiber film before and after carbonization

The preparation process of Zn/Co–N@PCNFs obtained from electrospun Zn/Co-ZIFs/PAN nanofibers is shown in Fig. 1. First, Zn/Co-ZIFs/PAN nanofibers were fabricated via an electrospinning method. Next, the as-obtained precursor nanofibers were carbonized directly at high temperature. The obtained carbon nanofibers were directly used as ORR electrocatalysts. Figure 1b, c shows the simulated cross section diagram and simulated molecular structure diagram, respectively, in different experimental stages. In the electrospinning process (I), the polymer chain of PAN wound around the Zn/Co-ZIF nanocrystals, forming an organic–inorganic compound system. During the carbonization process (II), the polymer chain of PAN formed a trapezoidal structure through the complex cyclic dehydrogenation reaction [40]. The skeleton of ZIF nanocrystals began to collapse in the carbonization process. During this process, the nitrogen atoms from the 2-methylimidazole and PAN polymer reconnected to the metal ions, making a uniform formation of active sites (metal nanoparticles, N_*x*_–C, and metal–N_*x*_–C) in the carbon nanofibers [41]. After carbonization (III), the N-doped graphite carbon layer was formed, which is also mainly embedded with cobalt nanoparticles and complex Co–N_*x*_–C catalytic activity sites. In addition, empty cavities formed from the position where the original Zn/Co-ZIF nanocrystals occupied before. This phenomenon suggests that the Zn/Co-ZIF nanocrystals not only serve as a doping agent but also serve as a pore-forming template. These continuous cavities form a porous structure, facilitating mass transfer during the catalytic process. Moreover, the large amount of Zn evaporation also plays a role in pore-forming, which enlarges the surface area and prevents the aggregation of cobalt nanoparticles. The digital photographs of the samples are shown in Fig. 1d, showing that the color of the nanofiber film changed from purple to black after carbonization, which indicates that the organic–inorganic composite system was converted into inorganic carbon nanofiber materials. Interestingly, Zn/Co–N@PCNFs-800 film still maintains flexible properties and a fibrous structure, which can facilitate its use as a self-supporting flexible device [42].

**Fig. 2:**
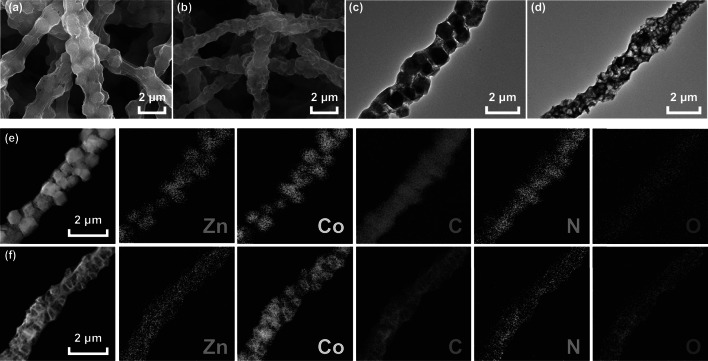
SEM images of the **a** Zn/Co-ZIFs/PAN nanofibers and **b** Zn/Co–N@PCNFs-800. TEM images of a single nanofiber **c** Zn/Co-ZIFs/PAN nanofiber and **d** Zn/Co–N@PCNFs-800. HR-TEM images and STEM elemental mappings of **e** a single Zn/Co-ZIFs/PAN nanofiber and **f** a single Zn/Co–N@PCNFs-800

The morphologies and structural features of the synthesized Zn/Co-ZIFs/PAN nanofibers and Zn/Co–N@PCNFs-800 were observed through SEM and TEM (Figs. 2 and S1). As shown in Fig. 2a, Zn/Co-ZIFs/PAN nanofibers have a raised rough surface. As the content of Zn/Co-ZIF nanoparticles increases, the roughness of the nanofiber surface increases (Fig. S1). However, the raised rough surface became folded after carbonization (Figs. 2b, S2, S3). In addition, the diameter of the nanofiber becomes smaller because of the volume contraction during the carbonization process. The TEM image (Fig. 2c) shows that the Zn/Co-ZIFs were successfully embedded into the PAN nanofibers. After the carbonization process, the porous empty cavities from the position of the original Zn/Co-ZIFs can be found in the carbon nanofibers, which is due to the collapse and volatilization of Zn/Co-ZIFs during the carbonization (Figs. 2d, S4). STEM elemental mapping was used to characterize the elemental distribution and change. As shown in Fig. 2e, the elements of Zn, Co, and N of Zn/Co-ZIFs/PAN are mainly concentrated in the Zn/Co-ZIFs. However, these elements are distributed on the nanofiber matrix uniformly after carbonization (Fig. 2f), which proves that Zn/Co-ZIFs play the roles of both self-sacrificing templates and doping agents.

**Fig. 3:**
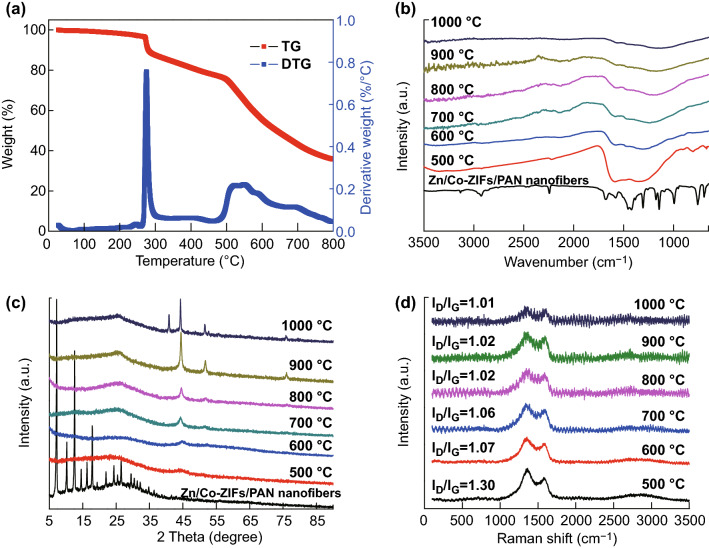
**a** TG and DTG curves of Zn/Co-ZIFs/PAN nanofibers. **b** FT-IR spectra, **c** XRD patterns, and **d** Raman spectra of Zn/Co-ZIFs/PAN nanofibers and their carbonized samples with different carbonization temperatures (500, 600, 700, 800, 900, and 1000 °C)

The carbonization process plays an important role in the performance of carbon-based ORR catalysts. Therefore, TG and DTG were used to monitor the carbonization behavior of Zn/Co-ZIFs/PAN nanofibers in N_2_ atmosphere (Fig. 3a). As we can see, the typical PAN degradation peak appeared at ~ 300 °C, which is likely associated with complex chemical reactions (dehydrogenation, cyclization, and cross-linking) during the stabilization process. The peak at ~ 550 °C for Zn/Co-ZIFs decomposition becomes broader [34], indicating that the Zn/Co-ZIF nanocrystals were embedded in the body of the nanofibers, which is consistent with the TEM images (Fig. 2c). The FT-IR spectra (Fig. 3b) were used to study the change in the functional groups during the carbonization process. The prominent organic functional group peaks gradually disappeared with increase in carbonization temperature, confirming that the organic–inorganic complex was converted into inorganic carbon materials. XRD patterns of the prepared samples are shown in Fig. 3c. The XRD patterns of the Zn/Co-ZIFs/PAN nanofibers are in good agreement with the simulated ZIFs, further suggesting that the PAN polymer matrix does not affect the ZIF crystal structure. The XRD patterns of different carbonized samples have four main peaks at 26°, 44.5°, 52°, and 76°, corresponding to the C (002), Co (111), Co (200), and Co (220) diffractions, respectively. Interestingly, a peak center at 41.3° for Co_2_C (002) appears at the higher carbonization sample temperature of 1000 °C. It is well known that the carbon graphitization and metal doping in the catalysts could enhance electronic conductivity and increase the number of active sites in electrocatalysts. Raman spectra were used to characterize the graphitization degree of the samples. All the Raman spectra display two prominent D-band and G-band peaks. Generally, the intensity ratio of the D-band to G-band (*I*_D_/*I*_G_) is used to estimate the disorder degree of the carbon material. As shown in Fig. 3d, the intensity ratios of *I*_D_/*I*_G_ vary from 1.30 to 1.01 with the increase of carbonization temperature from 500 to 1000 °C, suggesting that N doping generates a defective extrinsic structure on the carbon framework of carbon nanofibers. The defective structures can increase active sites and, thus, enhance electrochemical performance [43]. Furthermore, the decrease in *I*_D_/*I*_G_ indicates the increase of the degree of graphitic crystalline structure at higher carbonization temperatures. A balance is reached between doping and graphitization during carbonization.

**Fig. 4:**
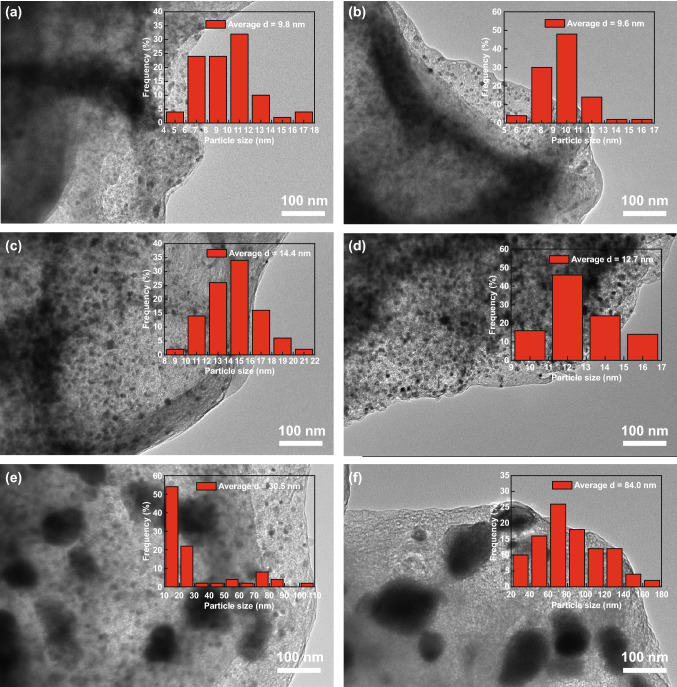
TEM images of the samples obtained at different carbonization temperature: **a** 500 °C, **b** 600 °C, **c** 700 °C, **d** 800 °C, **e** 900 °C, and **f** 1000 °C (Insets are the size distributions of metal nanoparticles)

TEM images of different samples were taken to further characterize the detail change in metal nanoparticles during the carbonization process (Figs. 4, S4). These TEM images and the size distribution of metal nanoparticles clearly show that the metal nanoparticles became larger in size with increase of carbonization temperature. Serious agglomeration of metal nanoparticles occurred when the carbonization temperature was 900 and 1000 °C, which does not assist the improvement of electrochemical performance because of the reduced surface area of active sites. Good distribution of doped-metal nanoparticles is important for excellent catalytic performance. As it can be seen from the results (Figs. 4, S4), the doped-metal nanoparticles were evenly distributed at carbonization temperatures of 700 and 800 °C.

**Fig. 5:**
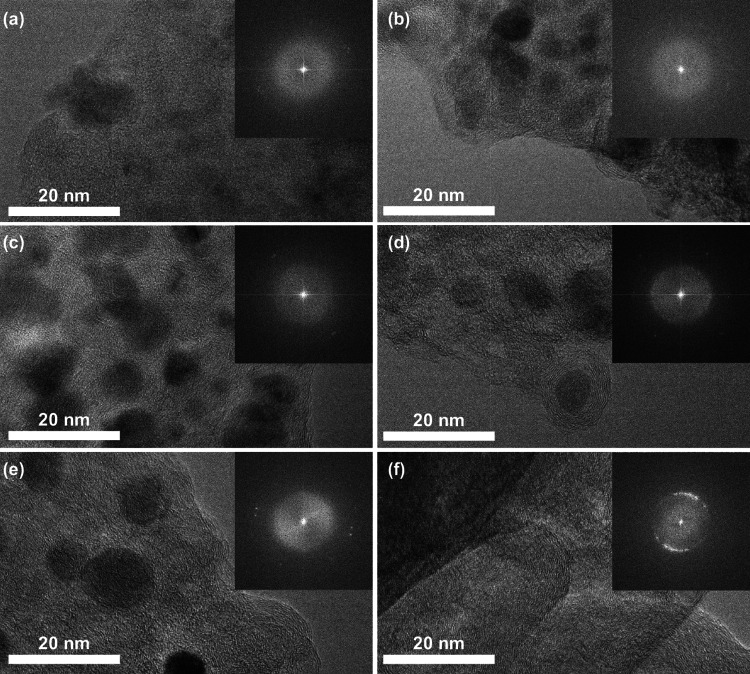
High-magnification HR-TEM images of the samples at different carbonization temperature: **a** 500 °C, **b** 600 °C, **c** 700 °C, **d** 800 °C, **e** 900 °C, and **f** 1000 °C (Insets are their corresponding SAED patterns)

Furthermore, changes in carbon graphite structure and metal crystallization were observed by high magnification HR-TEM images and the SAED patterns (shown in Fig. 5). The degree of carbon graphitization increased significantly with increase of carbonization temperature, and the crystal diffraction spots show the improvement in metal crystallization. This result is consistent with the Raman and XRD results. The graphitized network structure is helpful for improving the electron transfer rate in the process of catalysis. As it can be seen from Fig. 5, the metal nanoparticles were embedded in the graphitic carbon layer, which makes them difficult to detach from the substrate during the electrocatalytic process.

Doping is an important factor affecting electrochemical properties. XPS analyses were carried out to further characterize the change in elemental composition and chemical status of these carbonized samples. As shown in Fig. S5a, b, the elemental content of Zn, Co, and N decreased with increase of carbonization temperature, ranging from 500 to 1000 °C. The C/O ratio also increases with carbonization temperature, indicating that the conductivity gradually improved. The XPS and EDS results are consistent, which indicates that the composition of the material is uniform. The XPS high-resolution spectra of elemental Zn gradually disappeared when the temperature was above 900 °C, as elemental Zn in ZIFs easily evaporates (~ 900 °C), resulting in porous carbon structures during high-temperature treatment. The elemental content of cobalt increased first and then decreased with increase of carbonization temperature. This is due to the gradual doping of cobalt from the inside to the surface of nanofibers, which then evaporated at higher temperatures. The change in the content of elemental cobalt is consistent with the change in the nitrogen content, which imply the existence of Co–N_*x*_–C species. At the temperature of 800 °C, the sample has a relatively high content of cobalt and nitrogen. N atoms could incorporate into the graphene layers to replace carbon atoms at different sites during the carbonization process (above 700 °C), and in doing so, they were split into various binding energies in the XPS spectra: pyridinic-N 398.7 ± 0.3 eV, pyrrolic-N 400.4 ± 0.3 eV, and graphitic-N 401.4 ± 0.3 eV. It is worthy to note that carbons with pyridinic-N and pyrrolic-N at the edges of the graphene layers show higher charge mobility and better donor–acceptor properties than carbons with graphitic-N do [44].

**Fig. 6:**
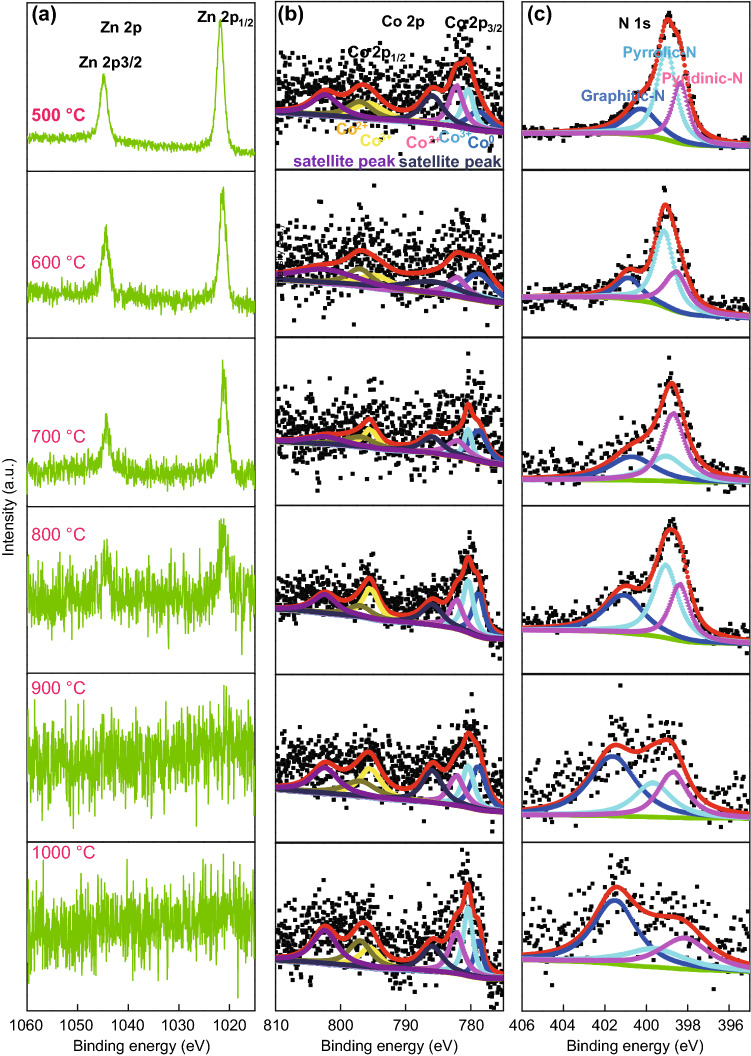
XPS high-resolution spectra of **a** Zn 2*p*, **b** Co 2*p*, and **c** N 1*s* levels at different carbonization temperatures

As shown in Fig. 6c, pyridinic-N and pyrrolic-N gradually converted into graphitic-N with carbonization temperature. There was also a partial transformation between pyridinic-N and pyrrolic-N in this process. Below the temperature of 700 °C, some pyrrolic-N was converted into pyridinic-N. Above the temperature of 700 °C, the content of pyridinic-N was gradually reduced. The content of pyrrolic-N increased from 700 to 800 °C and then gradually decreased above 800 °C. The total content of pyridinic-N and pyrrolic-N gradually decreased with the increase of temperature. At the temperatures of 700 and 800 °C, there was a relatively higher amount of pyridinic-N and pyrrolic-N. The binding energy of nitrogen increased with temperature, which proves the existence of Co–N_*x*_–C. The high-resolution Co 2p spectra of the samples are shown in Fig. 6b. It can be deconvoluted into five major peaks, at 778.8, 780.4, 782.2, 795.4, and 797.0 eV, corresponding to Co^0^, Co^3+^ 2*p*_3/2_, Co^2+^ 2*p*_3/2_, Co^3+^ 2*p*_1/2_, and Co^2+^ 2*p*_1/2_, respectively, and two shakeup satellite peaks at 785.9 and 802.4 eV [45, 46]. These peaks in the Co 2*p*_3/2_ XPS spectra also imply the existence of Co–N_*x*_–C species [47]. In addition, the peak of the metallic Co, located at the binding energy of 778.8 eV, confirms the presence of metallic cobalt nanoparticles [48]. This result is consistent with XRD and TEM results (Fig. 3c). The uniform dispersion of cobalt nanoparticles and the Co–N_*x*_–C activity sites guarantee the high electrocatalyst performance.

**Fig. 7:**
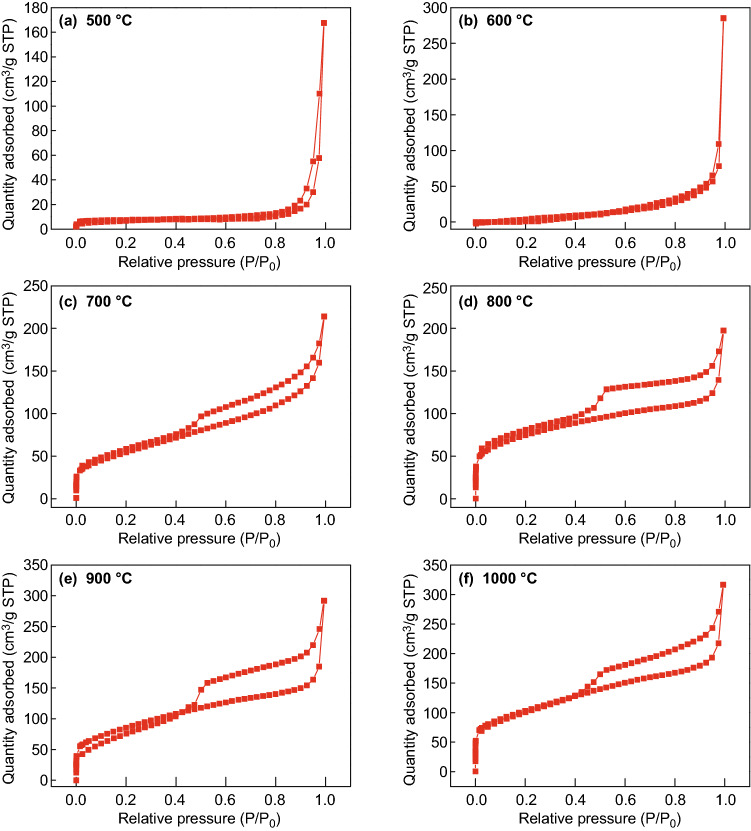
N_2_ adsorption–desorption isotherm of the samples at different carbonization temperature: **a** 500 °C, **b** 600 °C, **c** 700 °C, **d** 800 °C, **e** 900 °C, and **f** 1000 °C

Specific surface area is another important parameter that affects electrochemical performance. Large specific surface area may provide more active sites, especially for mesoporous structures. Macroporous structure also improves the mass transfer rate of electrolyte. The N_2_ adsorption–desorption isotherm and the corresponding pore-size distribution curves of the samples at different carbonization temperatures are shown in Figs. 7 and S6, respectively. The N_2_ adsorption–desorption isotherms demonstrate that the surface area increased with the increase of carbonization temperature. Above 700 °C, the N_2_ adsorption–desorption isotherm becomes a type IV isotherm with an H3-type hysteresis loop (*P*/*P*_0_ > 0.4), suggesting the mesoporous characteristic of the Zn/Co–N@PCNFs. The BET specific surface areas are 7.6, 25.5, 199.9, 265.2, 309.3, and 366.4 m^2^ g^−1^, at carbonization temperatures of 500, 600, 700, 800, 900, and 1000 °C, respectively. The pore-size distribution was calculated by the BJH method (shown in Fig. S6), and the average pore diameter of Zn/Co–N@PCNFs decreased gradually. Above 700 °C, many micropores appeared because of the volatilization of elemental zinc. At the same time, the average pore diameter is mesoporous below 5 nm. The features of high surface area and pore structure can be well maintained after the high-temperature treatment, which is the key for enhancing the transport of oxygen and electrolyte onto the catalyst surface for ORR. Although samples carbonized at 900 and 1000 °C have the highest specific surface area, the agglomeration of the nanoparticles and the reduction in the doping element led to less active sites.

**Fig. 8:**
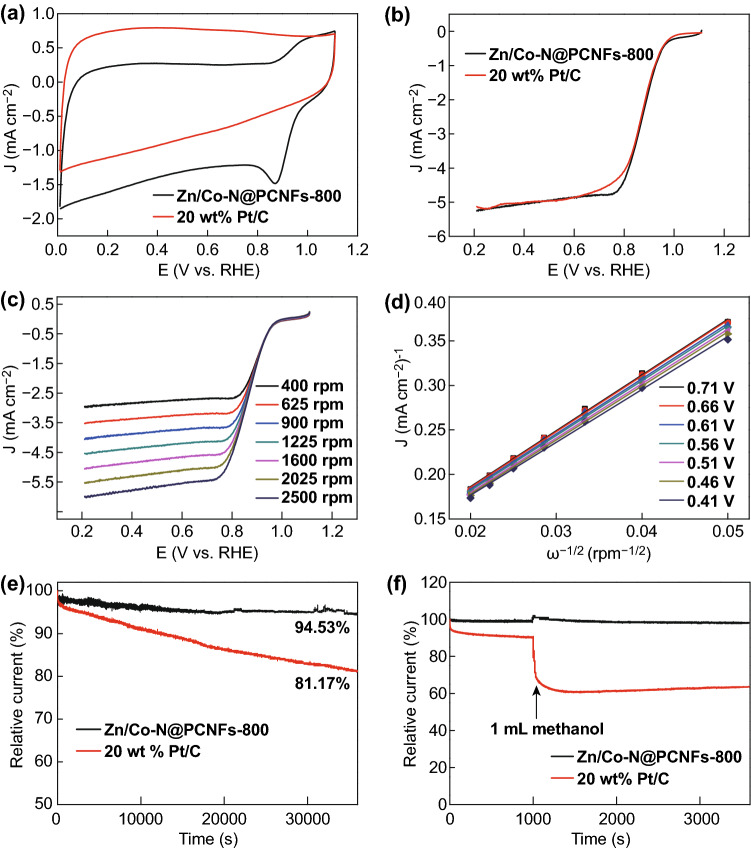
**a** CV curves of Zn/Co–N@PCNFs-800 in 0.1 M N_2_-saturated and O_2_-saturated KOH with a sweep rate of 50 mV s^−1^. **b** LSV curves for Zn/Co–N@PCNFs-800 and 20 wt% Pt/C in 0.1-M O_2_-saturated KOH electrolyte with a 10 mV s^−1^ and a rotation rate of 1600 rpm. **c** LSV curves of Zn/Co–N@PCNFs-800 at different rotation speeds from 400 to 2500 rpm. **d** *K*–*L* plots of Zn/Co–N@PCNFs-800 at different potentials. **e** Chronoamperometric response of Zn/Co–N@PCNFs-800 and 20 wt% Pt/C in 0.1 M O_2_-saturated KOH aqueous solution at 0.71 V versus RHE. **f** The durability test of Zn/Co–N@PCNFs-800 and 20 wt% Pt/C for methanol. The arrow indicates the introduction of 1 mL methanol

The electrocatalytic activity of the Zn/Co–N@PCNFs-800 was first examined by CV measurements in 0.1 M KOH solution. As shown in Fig. 8a, no obvious redox peak is observed for Zn/Co–N@PCNFs-800 in 0.1 M N_2_-saturated KOH solution. In contrast, a pronounced cathodic peak is clearly observed at 0.87 V (vs. RHE) in 0.1 M O_2_-saturated KOH solution. These results show the significant catalytic activity of oxygen reduction. The linear scan voltammetry (LSV) curves collected by the RDE demonstrate that Zn/Co–N@PCNFs-800 exhibited a better ORR activity (onset potential of 0.98 V vs. RHE), and half-wave potential (0.89 V vs. RHE). Zn/Co–N@PCNFs-800 also has larger diffusion-limited currents than the 20 wt% Pt/C has. For the other samples, the electrochemical performance first increased and then decreased with increase of carbonization temperature (Fig. S7). The excellent catalytic activity of Zn/Co–N@PCNFs-800 may result from the synergistic effect of a small amount of zinc-doping Co–N_*x*_–C species, uniform dispersion of Co nanoparticles and N dopants, high surface area, distinct conductive carbon nanofibers, and hierarchical pore structure [49, 50]. RDE measurements were conducted at various rotating speeds to investigate the kinetic parameters of Zn/Co–N@PCNFs-800 (Fig. 8c). With the increase in rotational speed, the diffusion current increased uniformly, which proves that the ORR process is well controlled by oxygen diffusion. The Koutecky–Levich (K–L) equation was used to analyze the kinetic parameters. The good linearity of the corresponding K–L plots suggested the first-order reaction kinetics toward the concentration of dissolved oxygen and at various potentials from 0.71 to 0.41 V (Fig. 8d). The electron transfer numbers at various potentials were calculated based on the K-L equations, and the obtained electron transfer number is about 3.88 from 0.71 to 0.41 V (V vs. RHE), indicating a direct four-electron oxygen reduction process (Fig. S8). Apart from the excellent ORR activity, Zn/Co–N@PCNFs-800 also exhibited much better stability and methanol tolerance as compared with the 20 wt% Pt/C. Chronoamperometric measurement at a voltage of 0.71 V recorded a greater than 94.53% current retention for a continuous 36,000 s operation (Fig. 8e). For comparison, 20 wt% Pt/C showed an significant activity decay with a less than 81.17% retention under the same testing conditions. In addition, Zn/Co–N@PCNFs-800 exhibited excellent resistance against methanol crossover. As shown in Fig. 8f, after the injection of 1 mL methanol, a slight change in the current occurred for our catalyst, while the cathodic current of 20 wt% Pt/C decreased sharply. To further test its electrochemical performance, the OER performance of Zn/Co–N@PCNFs-800 was tested (Fig. S9) in 0.1 M KOH solution. The Zn/Co–N@PCNFs-800 exhibited similar OER activity and more stable cyclic voltammetry, compared with the commercial RuO_2_ catalyst.

**Fig. 9:**
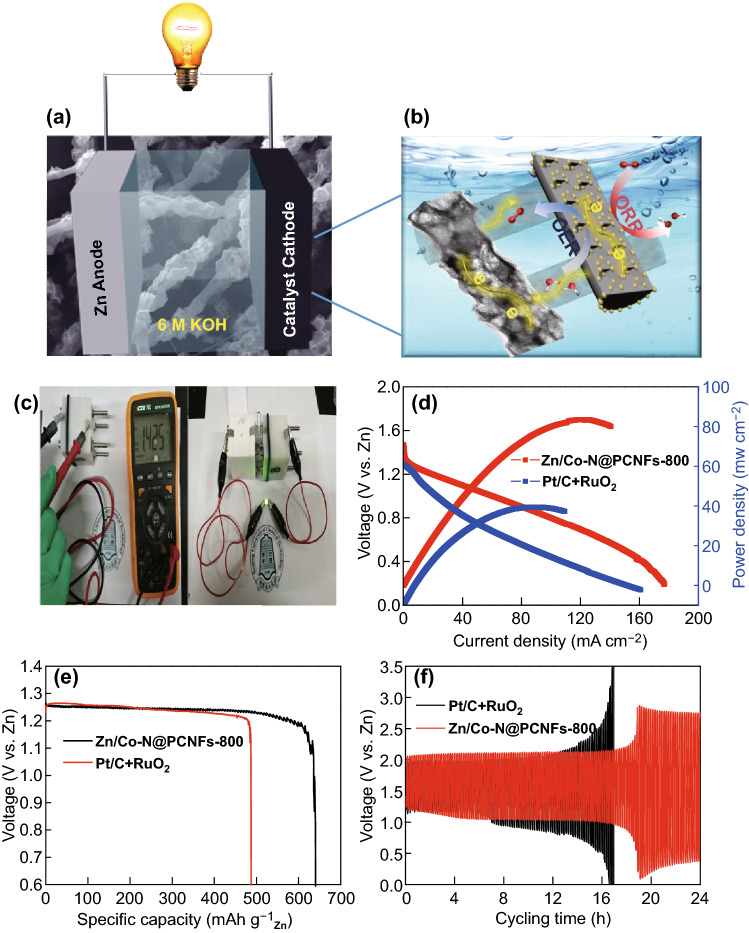
**a** Schematic representation of the basic configuration of a two electrode Zn–air battery by coupling the Zn electrode with an air electrode to execute ORR and OER in 6 M KOH solution as the electrolyte. **b** Catalytic mechanism diagram of cathode about Zn/Co–N@PCNFs-800. **c** Photographs of a green LED (1.8 V) powered by two Zn–air batteries integrated in series. **d** Discharge polarization and power density curves of the Zn–air batteries using Zn/Co–N@PCNFs-800 and 20 wt% Pt/C + RuO_2_ as ORR catalysts (mass loading of 1.2 mg cm^−2^). **e** Specific capacities for the Zn–air battery using Zn/Co–N@PCNFs-800 and 20 wt% Pt/C + RuO_2_ as an ORR catalyst, which was regularized with consumed Zn mass. **f** Galvanostatic discharge and charge cycling curves at 10 mA cm^−2^ with each cycle for 10 min (5 min charge and 5 min discharge) of rechargeable Zn–air batteries with the Zn/Co–N@PCNFs-800 and 20 wt% Pt/C + RuO_2_ as the cathode catalyst

A homemade Zn–air battery was further assembled to demonstrate the cell performance. The catalysts covering the PTFE-treated carbon fiber paper, 6 M KOH, and a zinc foil acted as the air cathode, electrolyte, and anode, respectively (Fig. 9a). Figure 9b shows the catalytic mechanism diagram of cathode about Zn/Co–N@PCNFs-800. The carbon nanofiber matrix provides a good electron conduction transfer channel, the porous structure provides a mass transfer channel, and the uniform dispersion of metal nanoparticles provides sufficient activity sites. Two zinc–air batteries based on Zn/Co–N@PCNFs-800 catalysts were integrated in series to power a green light-emitting diode (LED, 1.8 V). An open-circuit voltage of ca. 1.425 V was observed when the battery was loaded with Zn/Co–N@PCNFs-800 in Fig. 9c. Figure 9d presents polarization and power density curves. Notably, the voltage decreases with the increase of current density, with the peak power density of Zn/Co–N@PCNFs-800 at 83.5 mW cm^−2^ for a current density of 124.5 mA cm^−2^, higher than that of 20 wt% Pt/C + RuO_2_ (44.9 mW cm^−2^ for a current density of 84.2 mA cm^−2^), further highlighting the key role of interconnected hierarchical porous structures in the fast electron/ion pathway and gas diffusion [51, 52]. Zn/Co–N@PCNFs-800-based Zn–air batteries possess a specific capacity of 640.3 mAh g^−1^ Zn when normalized to the mass of consumed Zn at a discharge density of 10 mA cm^−2^ (Fig. 9e). Figure 9f shows the galvanostatic discharge and charge cycling curves of rechargeable Zn–air batteries at 10 mA cm^−2^ with 10 min cycles (5 min charge and 5 min discharge), with the Zn/Co–N@PCNFs-800 and 20 wt% Pt/C + RuO_2_ as the cathode catalyst. Zn/Co–N@PCNFs-800 exhibited excellent reversibility and better cycling life (more than 18 h) than the commercial 20 wt% Pt/C + RuO_2_ (~ 16 h) catalysts.

## Conclusions

In summary, flexible, porous, and well-dispersed metal–heteroatom-doped carbon nanofibers were prepared by a direct high-temperature carbonization approach using electrospun Zn/Co-ZIFs/PAN nanofibers as the precursor. The flexible porous bimetal–heteroatom-doped carbon nanofibers exhibited the excellent ORR electrocatalytic activity, superior stability, and methanol tolerance under 0.1 M KOH solution, which can be ascribed to the synergistic effect of Co–N_*x*_ species, uniform dispersions of Co nanoparticles and N dopants, high surface area, distinct conductive curving of carbon nanofibers, as well as the hierarchical pore structure. The excellent ORR performance was also demonstrated in a homemade rechargeable zinc–air battery. In addition, this Zn/Co–N@PCNFs-800 film exhibited good flexibility, which could be applied to flexible devices. Our work illustrates the great potentials of hybrid porous carbon nanofiber materials as ORR and OER electrocatalysts. We hope that this work can spark interests in developing multi-functional electrocatalysts toward application in renewable energy technologies.

## Electronic supplementary material

Below is the link to the electronic supplementary material.

Electronic supplementary material 1 (PDF 734 kb)
