# Supplementary material for: Flexible, Porous, and Metal–Heteroatom-Doped Carbon Nanofibers as Efficient ORR Electrocatalysts for Zn–Air Battery
Source: Nanomicro Lett. 2019 Jan 19;11:8. doi: 10.1007/s40820-019-0238-4 (PMC7770869; doi:10.1007/s40820-019-0238-4)
Supplement: Supplementary file 1 — Electronic supplementary material 1 (PDF 734 kb) [file 40820_2019_238_MOESM1_ESM.pdf]

Supporting Information for

Flexible, Porous, and Metal-Heteroatom-doped Carbon Nanofibers as Efficient ORR Electrocatalysts for Zn-air Battery

Qijian Niu[1, 2, #], Binling Chen[3,*, #], Junxia Guo[1, 2], Jun Nie[1, 2], Xindong Guo[1, 2], Guiping Ma[1, 2, *]

[1]Key Laboratory of carbon fiber and functional polymers, Ministry of Education, Beijing University of Chemical Technology, Beijing 100029, People's Republic of China

[2]State Key Laboratory of Chemical Resource Engineering, Beijing University of Chemical Technology, Beijing 100029, People's Republic of China

[3]College of Engineering, Mathematics and Physical Sciences, University of Exeter, Exeter, EX4 4QF, UK

[#]Qijian Niu and Binling Chen have contributed equally to this work

*Corresponding authors. E-mail: magp@mail.buct.edu.cn (Guiping Ma); B.Chen@exeter.ac.uk (Binling Chen)

Supplementary Figures

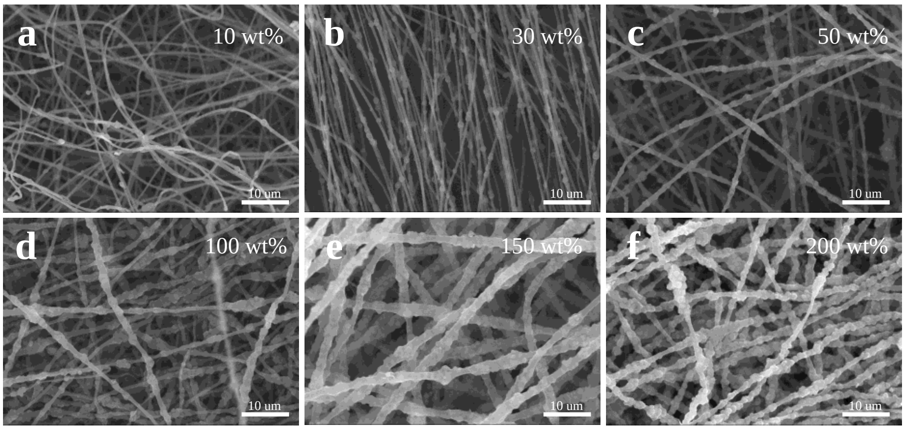

Fig. S1 SEM images of the Zn/Co-ZIFs/PAN nanofibers with different content Zn/Co-ZIFs loading: **a** 10 wt%, **b** 30 wt%, **c** 50 wt%, **d** 100 wt%, **e** 150 wt%, and **f** 200 wt%

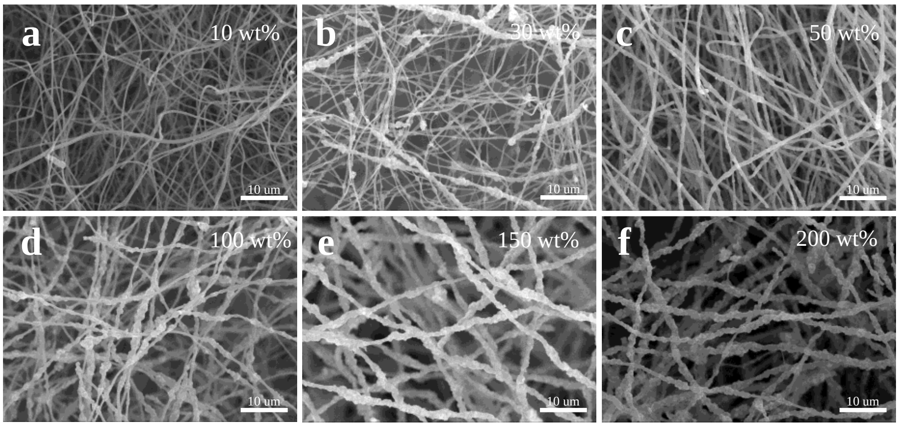

Fig. S2 SEM images of the Zn/Co-ZIFs/PAN nanofibers with different content Zn/Co-ZIFs loading at the carbonization temperature of 800 ºC: **a** 10 wt%, **b** 30 wt%, **c** 50 wt%, **d** 100 wt%, **e** 150 wt%, and **f** 200 wt%

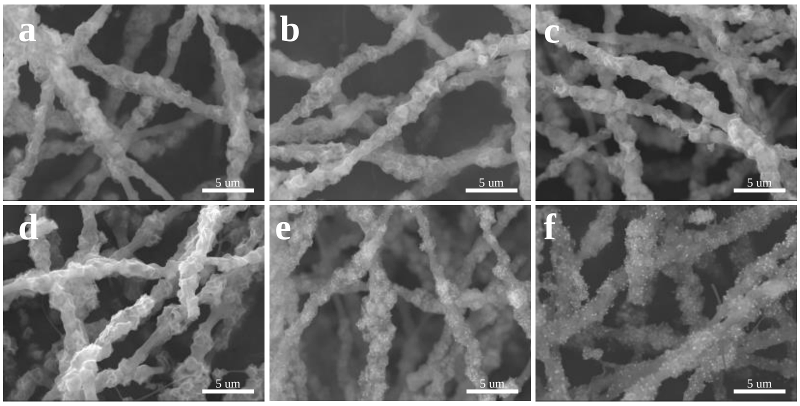

Fig. S3 SEM images of the Zn/Co-ZIFs/PAN nanofibers with 200 wt% Zn/Co-ZIFs loading at different carbonization temperature: **a** 500ºC, **b** 600 ºC, **c** 700 ºC, **d** 800 ºC, **e** 900 ºC, and **f** 1000 ºC

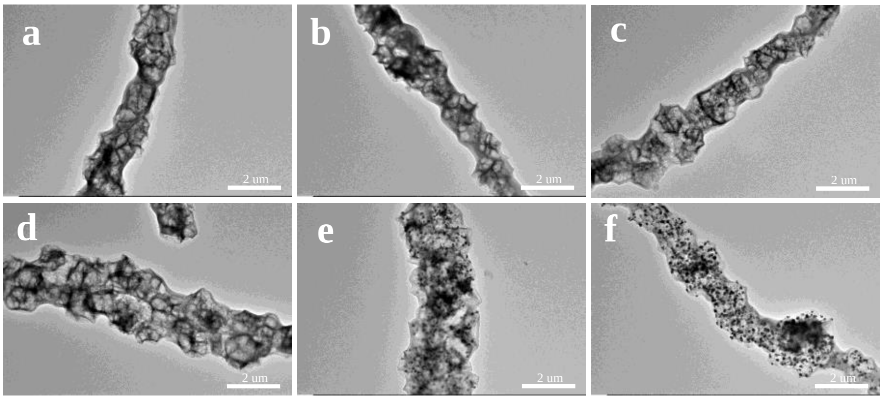

Fig. S4 TEM images of the Zn/Co-ZIFs/PAN nanofibers with 200 wt% Zn/Co-ZIFs loading at different carbonization temperature: **a** 500 ºC, **b** 600 ºC, **c** 700 ºC, **d** 800 ºC, **e** 900 ºC, and **f** 1000 ºC

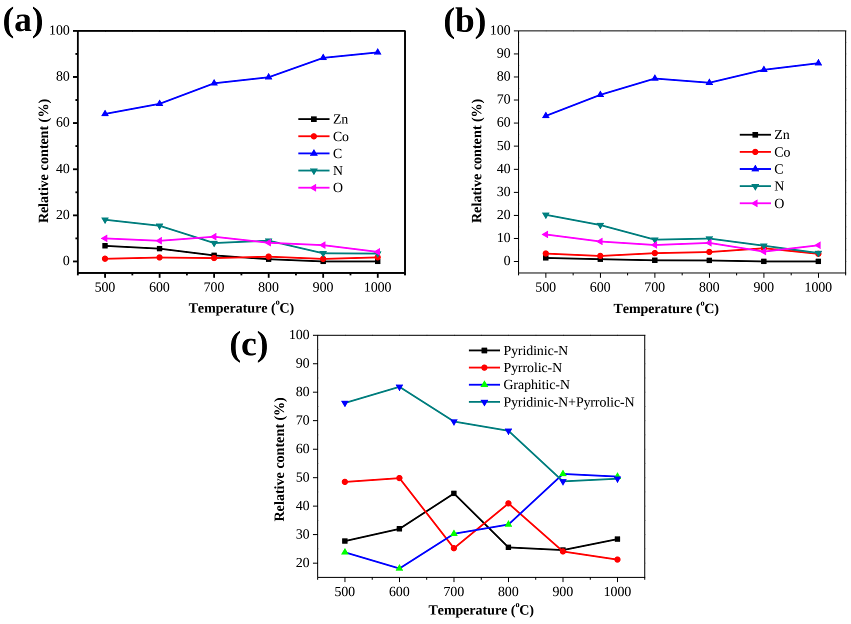

Fig. S5 The element content of the Zn/Co-ZIFs/PAN nanofibers with 200 wt% Zn/Co-ZIFs loading at different carbonization temperature: **a** XPS results, **b** EDS results, **c** the content of different forms of N at different carbonization temperature

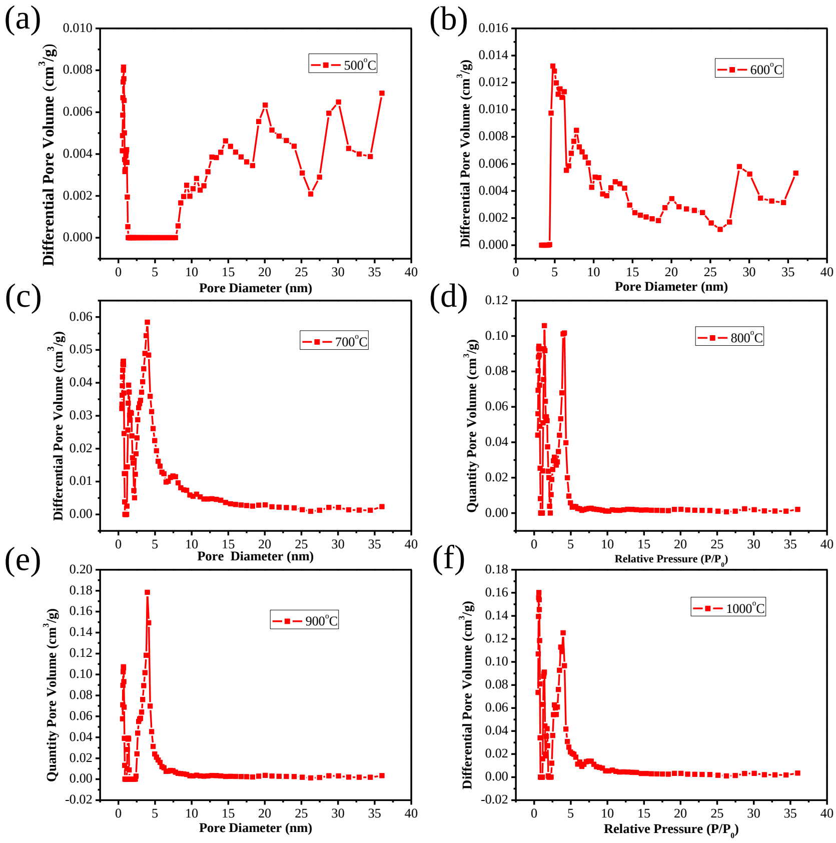

Fig. S6 The pore size distribution of the samples at different carbonization temperature: **a** 500 °C, **b** 600 °C, **c** 700 °C, **d** 800 °C, **e** 900 °C, and **f** 1000 °C

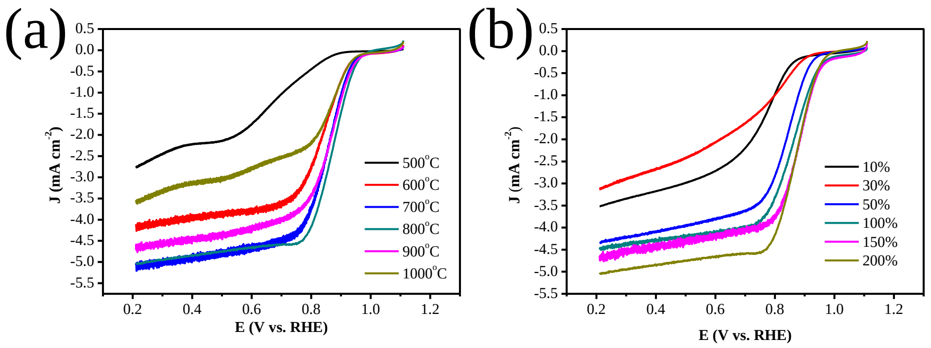

Fig. S7 a The LSV curves for different samples with different Zn/Co-ZIFs contents; **b** The LSV curves for different samples of the Zn/Co-ZIFs/PAN nanofibers with 200 wt% Zn/Co-ZIFs loading at different carbonization temperature in 0.1 M KOH electrolyte with a 10 mV s^{-1} and a rotation rate of 1600 rpm

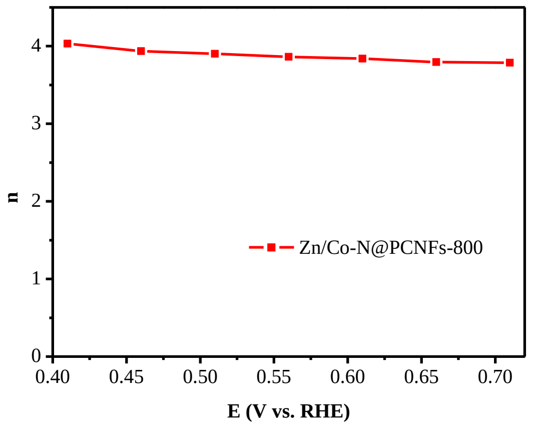

Fig. S8 The electron transfer number of Zn/Co-N@PCNFs-800 as an ORR electrocatalysts in 0.1 M KOH

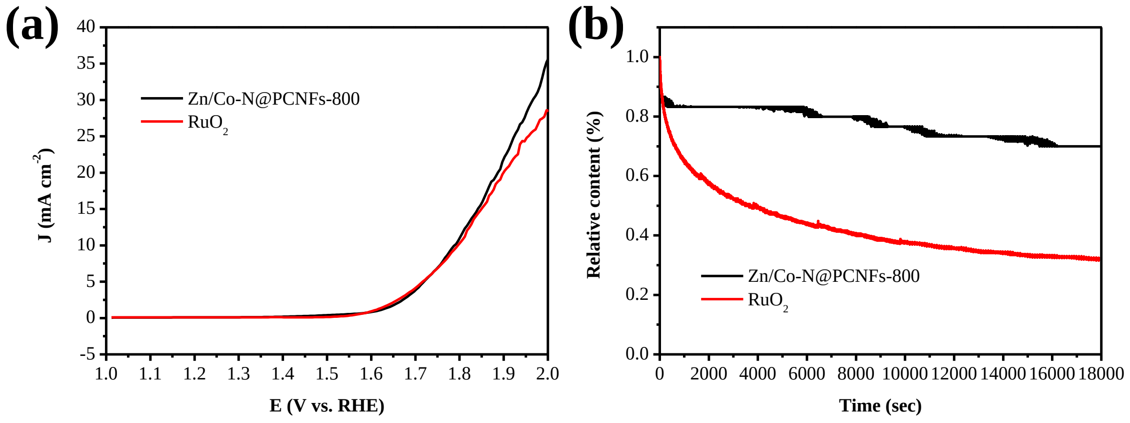

Fig. S9 a LSV curves of Zn/Co-N@PCNFs-800 and RuO_2; **b** Chronoamperometric response for Zn/Co-N@PCNFs-800 and RuO_2 at 1.61 V vs. RHE
